# Brain imaging findings in lipoid proteinosis (Urbach-Wiethe disease)

**DOI:** 10.1016/j.radcr.2025.06.085

**Published:** 2025-07-19

**Authors:** Athanasios Tsochatzis, Nikolaos-Achilleas Arkoudis, Varvara Pantoleon, Panagiotis Toulas, Dimitrios Filippiadis, Georgios Velonakis

**Affiliations:** a Department of CT and MRI, Diagnostic and Therapeutic Centre ‘Hygeia’, Athens, Greece; b Research Unit of Radiology and Medical Imaging, Second Department of Radiology, Medical School, National and Kapodistrian University of Athens, Athens, Greece; c Second Department of Radiology, School of Medicine, Attikon University General Hospital, National and Kapodistrian University of Athens, Athens, Greece

**Keywords:** Calcifications, Epilepsy, Imaging, Lipoid proteinosis, Neuroradiology, Radiology, Urbach-Wiethe disease

## Abstract

We present neuroimaging and skin findings of Urbach-Wiethe disease (lipoid proteinosis) in 2 adult patients. Lipoid proteinosis is a rare, autosomal recessive disease that primarily affects the skin, the upper respiratory tract, and the central nervous system (CNS). The first patient (a 37-year-old female) was referred due to the new onset of bilateral temporal lobe epilepsy. She had a family history and typical skin manifestations of the disease. She had been diagnosed with the disease in childhood. The second patient (a 42-year-old male) was referred for an MRI due to episodes of migraine and panic attacks without a previous diagnosis of the disease. Bilateral mesial temporal lobe calcifications, especially in the amygdalae, were found on MRI and CT in both patients. Patients may be referred for imaging due to a variety of neurological symptoms, even without prior knowledge of the disease; thus, radiologists should be aware of the imaging manifestations of lipoid proteinosis.

## Introduction

Urbach-Wiethe disease (lipoid proteinosis) is a rare, autosomal recessive disease that was initially described by Urbach and Wiethe in 1929 as “hyalinosis cutis et mucosae” [1]. The causal genetic defect is thought to be loss-of-function mutations in the extracellular matrix protein 1 gene (ECM1) on chromosome 1q21 [2,3]. This mutation results in the deposition of periodic acid-Schiff-positive (PAS) hyaline material in the dermis, mucous basement membranes, blood vessels, and adnexal epithelia [3].

The disease primarily affects the skin, which along with the upper respiratory tract and the central nervous system constitute the most commonly affected regions of the body [3,4]. Manifestations reflecting central nervous system (CNS) involvement vary widely, including epilepsy and psychotic symptoms [5,6]. We present 2 cases of Urbach-Wiethe disease with initial brain imaging findings depicted in adulthood due to the emergence of new symptoms.

## Case reports

### Patient 1

**Fig. 1:**
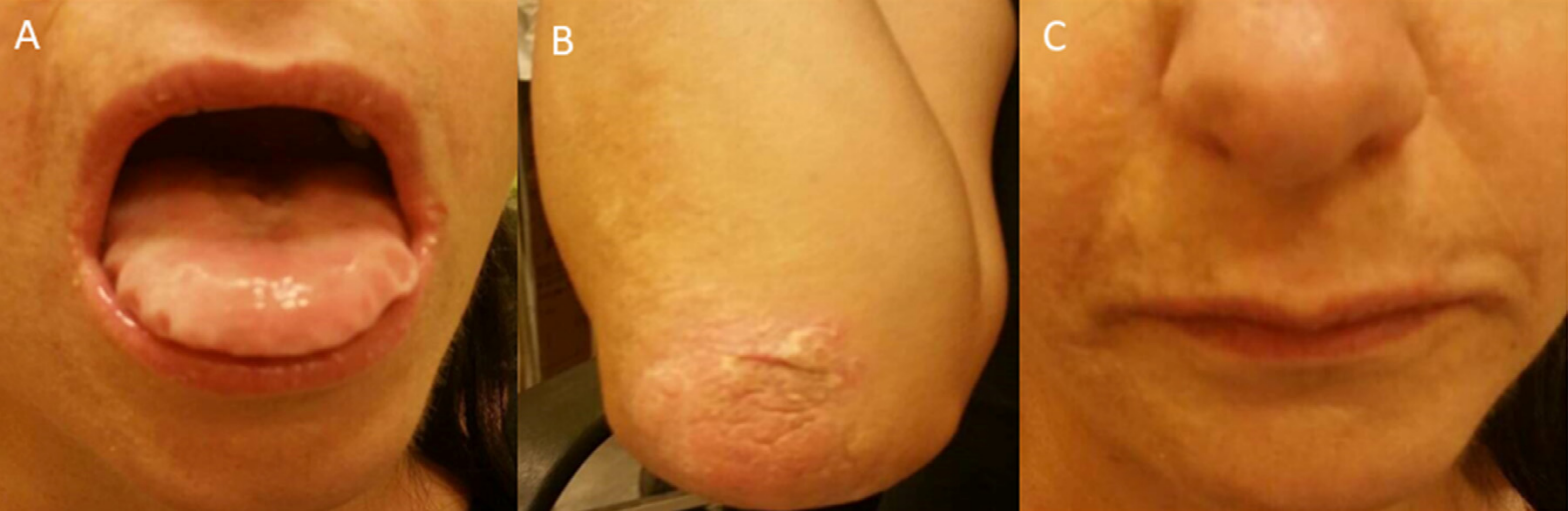
Photographs of a 37-year-old female patient with lipoid proteinosis demonstrate thickened, white oral mucosa (A), the formation of waxy plaques at elbows (B), and skin thickening most evident on the face (C).

A 37-year-old female patient with neuropsychiatric sequelae, including psychotic symptoms, was referred from the neurology outpatient department for an MRI due to the onset of bilateral temporal lobe epilepsy. The patient had a family history of Urbach-Wiethe disease (an affected sibling), and she had therefore been histologically and genetically diagnosed with the disease since the age of 2. However, up until this time, she had never undergone imaging. On physical inspection, she demonstrated thickened white oral mucosa (Fig. 1A), dermatological manifestations such as the development of multiple waxy plaques, particularly at sites of friction (eg, elbows) (Fig. 1B), scarring of the skin (Fig. 1C), and alopecia. Moreover, she had multiple papules along the eyelid margins (known as moniliform blepharosis). She also had a history of progressive hoarseness.

**Fig. 2:**
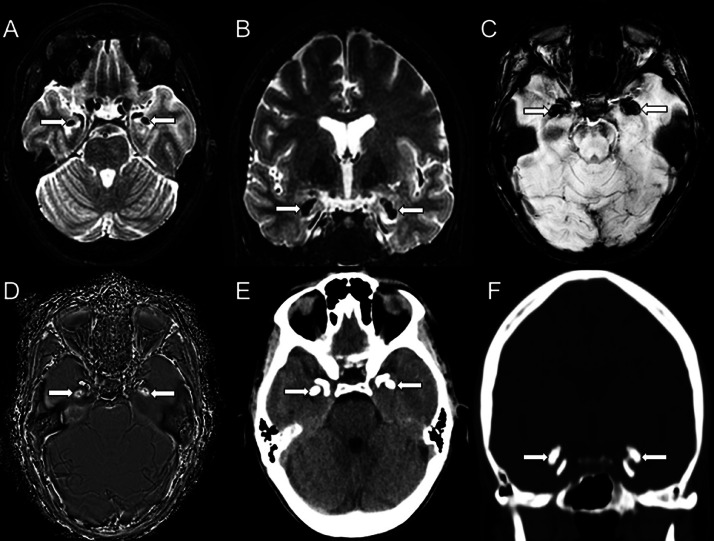
Axial (A), coronal (B) T2-weighted, and SWI (C) images reveal signal loss (arrows) consistent with calcifications on filtered-phase images (D) in the amygdalae and the head of the hippocampi in a 37-year-old female patient with lipoid proteinosis. CT axial (E) and coronal (F) images confirm the presence of calcifications (arrows).

Routine laboratory testing, including complete blood count, electrolytes, liver and renal function, and thyroid profile, was within normal limits. ESR and CRP were also unremarkable (Normal values: WBC 4-10 K/µL, hemoglobin 12-16 g/dL, creatinine 0.6-1.2 mg/dL, TSH 0.4-4.0 µIU/mL).T1-weighted and T2-weighted sequences demonstrated bilateral, hypointense lesions in the mesial temporal lobes (Fig. 2A, B). Susceptibility-weighted imaging (SWI) revealed symmetrical signal loss in the amygdalae and on the head of the hippocampi (Fig. 2C). Findings were consistent with calcifications on SWI-filtered phase imaging (Fig. 2D). Finally, supplementary imaging with CT showcased symmetrical, coma-shaped calcifications in the aforementioned regions, hence further validating the SWI-filtered phase imaging findings (Fig. 2E, F).

Management included initiation of antiepileptic therapy with levetiracetam 500 mg twice daily. She responded well, with a reduction in seizure frequency over 6 months of follow-up. No surgical intervention was deemed necessary. Neuropsychiatric support was also initiated.

### Patient 2

A 42-year-old male was referred for an MRI due to the new onset of episodes of migraine and panic attacks. He had no known prior neurological or psychiatric conditions. His medical history was otherwise unremarkable, and he had no history of surgical procedures. The patient described a gradual onset of anxiety episodes characterized by palpitations, a sense of impending doom, and light-headedness, often followed by headaches. He also reported mild hoarseness since adolescence, which had remained unexplored.

**Fig. 3:**
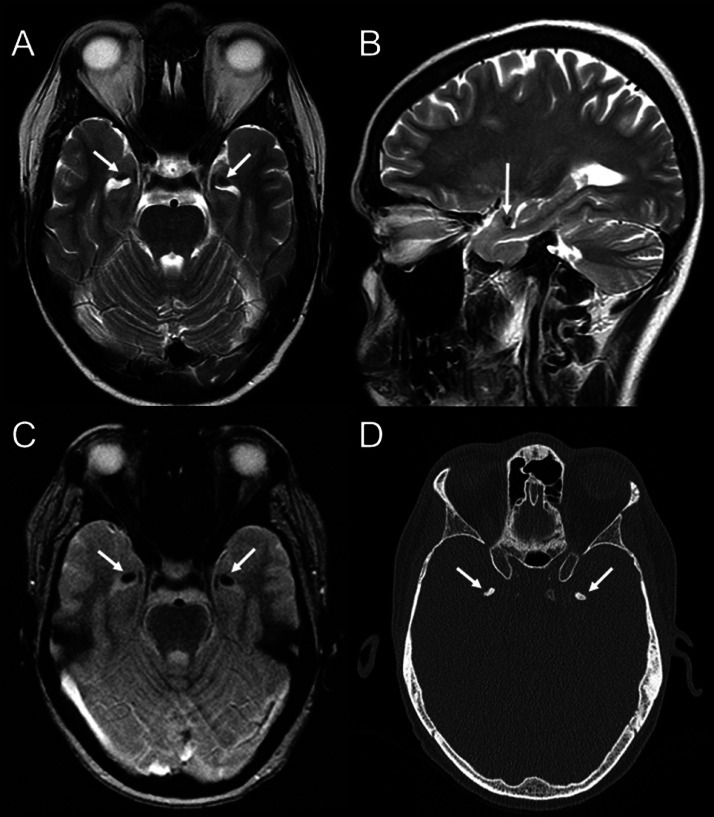
Axial (A), sagittal (B) T2-weighted, and axial T2* (C) images demonstrate bilateral hypointensities (arrows) in amygdalae in a 42-year-old male patient with lipoid proteinosis. (D) An axial CT image confirms calcifications.

No skin manifestations were observed apart from waxy plaques on the axillae, which the initial clinical examination overlooked. The patient had ignored his light hoarseness and had failed to seek medical attention. There was no family history of lipoid proteinosis or similar symptoms. Laboratory results were within normal limits, including serum calcium, phosphate, liver and renal function tests, and inflammatory markers. MRI revealed bilateral T1 and T2 hypointensities in the amygdalae (Fig. 3A and B). Signal loss was seen in the T2* sequence (Fig. 3C). CT verified the presence of bilateral amygdalae calcifications (Fig. 3D). Genetic testing confirmed the diagnosis of lipoid proteinosis.

The patient was managed conservatively with psychological counseling for panic symptoms and initiated on low-dose SSRIs. Neurological symptoms remained stable during follow-up. No antiepileptic or surgical therapy was required.

### Imaging technique

The MRIs were performed with a Philips 3T Achieva TX MRI scanner (Philips, Best, the Netherlands) with an 8-channel head coil. Sequences included T1- and T2-weighted imaging, diffusion-weighted imaging (DWI), susceptibility-weighted imaging (SWI), GRE T2*-weighted imaging, 3D FLAIR and 3D T1-weighted images following an intravenous contrast injection. The CT scans were performed on a Philips Brilliance™ 64 CT scanner.

## Discussion

We reported 2 cases of lipoid proteinosis in adult patients. The imaging hallmark for this disease is bilateral symmetric horn-shaped mesial temporal lobe calcifications, typically affecting the amygdalae, which were depicted in both cases presented. Calcifications are more evident with longer disease durations. Other frequently affected sites include the hippocampus, parahippocampal gyrus, and striatum [5]**.** In the absence of brain calcifications, imaging findings may be unremarkable [7].

For a long time, CT has been appraised as the method of choice for identifying calcifications, considering how it may be challenging to ultimately pinpoint their presence on MRI [8]. However, SWI can identify small amounts of hemorrhage/blood products, or calcium, both of which may be undetected on other MRI sequences. Distinguishing between the two is feasible on filtered-phase images because diamagnetic and paramagnetic compounds affect phase differently, resulting in opposite signal intensity [8]. In our first patient, filtered-phase images verified the presence of calcifications. To our knowledge, the implementation of SWI and filtered phase images has not been formerly described in Urbach-Wiethe disease and could imply that CT may not be necessary for the detection of calcifications in such patients.

CNS involvement is seen in 50%-75% of affected individuals [9,10]. Infiltration primarily occurs around the hippocampal capillaries, causing wall thickening, which later progresses to perivascular deposition of calcium [5]. Microscopic findings are comprised of amorphous calcifications encompassed by gliotic tissue and calcified, thickened capillary walls [7]. Manifestations reflecting CNS involvement vary widely, including temporal lobe epilepsy, migraine, intellectual disability, depression, anxiety, panic attacks, disturbances in memory and decision-making, and psychotic symptoms [5]. While panic attacks are typically considered functional or psychiatric in origin and are not standard indications for neuroimaging, there are clinical scenarios in which imaging may be considered useful. In our second case, the patient presented with atypical features, including late-onset panic-like episodes accompanied by migraines and subtle cognitive complaints, which made exclusion of a structural cause desirable. In similar settings, particularly in the presence of neurological symptoms with atypical features, imaging may assist in excluding organic pathology.

Amygdalae play a key role in mediating emotions, especially fear recognition associated with possible danger and threat [6]. Our first patient had a family history of Urbach-Wiethe disease. Her brother was also suffering from lipoid proteinosis, and he had demonstrated a history of careless behavior, a fact that had led to his passing away in a motorcycle accident due to his reckless driving. None of the cases presented herein demonstrated any significant abnormalities related to fear recognition.

Infiltration of the larynx with hyaline material may cause hoarseness, which can be present at birth and may therefore constitute the initial disease presentation. However, it may also present at later ages and can progress with time [3,11]. Both patients had hoarseness; however, the second patient ignored this unspecific symptom and therefore failed to seek medical attention. Other upper respiratory tract manifestations include infiltration of the tongue, infiltration of the oral mucosa causing xerostomia and dysphagia, and diffuse infiltration of the pharynx and larynx, which could potentially but infrequently lead to respiratory obstruction [3].

Dermatological involvement is the most common clinical manifestation. Skin is greatly prone to damage, and diffuse infiltration causes gradual thickening, leading to the formation of yellowish papules and chicken pox-like scars, mainly affecting the face and extremities [3]. Beaded papules are usually formed on the eyelid margins—a pathognomonic finding known as moniliform blepharosis seen in 50% of the patients [4]. On the extremities, skin infiltration can be seen as groups of warty plaques on the axillae, elbows, and generally at sites of friction [3]. Alopecia may also eventually develop [4]. Reported ocular manifestations include dry eyes, glaucoma (open angle), retinitis pigmentosa, uveitis, and subluxation of the lens [12].

Patients suffering from this disease have a near-normal life span, although currently there is no known definitive cure [11]. However, symptomatic treatment may be recommended (eg, antiepileptic drugs when clinically manifesting with epilepsy).

### Differential diagnosis

On imaging, especially CT and SWI, bilateral, symmetric, horn-shaped amygdalae calcifications (which were present in both our cases) are the hallmark of lipoid proteinosis, are more evident with longer duration of the disease [5,7], and remain highly suggestive of it, especially when accompanied by relevant clinical findings. Nonetheless, it is useful to acknowledge that the bilateral amygdalae calcifications seen in lipoid proteinosis may on some occasions have to be differentiated from other causes of mesial temporal lobe calcifications. Neurocysticercosis, a parasitic infection, can produce calcified granulomas, which occasionally may involve the temporal lobes, though typically with a more random distribution [13]. Mesial temporal sclerosis (MTS), commonly associated with epilepsy, may rarely present with calcifications, which would be suggestive of prior neurocysticercosis [14]. The primary imaging feature of MTS would be hippocampal atrophy and signal alterations [15]. Metabolic disorders (ie, hypoparathyroidism) or conditions such as Fahr disease, can result in intracranial calcifications, but these will usually involve the basal ganglia rather than the mesial temporal lobes [16]. Additional differential considerations include calcified gliomas, healed herpes encephalitis, and other postinfectious or neoplastic processes [16].

### Diagnostic pathway

Finaly, to consolidate and clarify the diagnostic pathway, the diagnosis of lipoid proteinosis is based on a combination of clinical, histopathological, genetic, and radiological findings. Clinically, characteristic features include early-onset hoarseness, beaded eyelid papules (moniliform blepharosis), and skin scarring. Histopathology reveals periodic acid–Schiff (PAS)-positive hyaline material deposited in the dermis and mucous membranes. Genetic confirmation is achieved through identification of ECM1 gene mutations on chromosome 1q21. Imaging (particularly MRI with SWI and CT) can reveal the disease’s hallmark feature, which is bilateral symmetric calcifications in the amygdalae and other mesial temporal structures. While not essential for diagnosis, these findings are highly suggestive when correlated with clinical signs and can elicit further tests to confirm diagnostic suspicions.

### Role of imaging

Although lipoid proteinosis is confirmed genetically via ECM1 mutation analysis, CNS imaging may have a potential role in assessing disease extent and symptom correlation. As previously mentioned, the presence of bilateral amygdalae calcifications has been associated with longer disease duration and is often linked to specific neuropsychiatric manifestations such as temporal lobe epilepsy, emotional dysregulation, and memory impairment [5,7]. In this context, CT and SWI can confirm the diagnosis and possibly guide symptomatic management. On the contrary, the absence of calcifications cannot rule out the disease but may suggest a milder CNS phenotype or an earlier disease stage [5,7]. Therefore, imaging findings can contribute meaningful prognostic and clinical information beyond genetic testing alone.

## Conclusion

We reported 2 adult patients with lipoid proteinosis demonstrating bilateral, symmetric amygdalae calcifications, which constitute the imaging hallmark of the disease. Radiologists should be aware of this imaging pattern considering how patients may be referred due to a variety of often unspecific neurological symptoms without a previous diagnosis of the disease, even in adulthood.

## Patient consent

The patients provided written, informed consent for the publication of their case, including relevant clinical details and any accompanying images.

## Author contributions

All authors designed and prepared the manuscript. The first draft of the manuscript was written by AT, NAA and GV, and all authors commented on previous versions of the manuscript. VP assisted with data collection. PT and DF assisted with supervision, writing review and editing. All authors read and approved the final manuscript. Authors NAA and AT contributed equally to all aspects of this manuscript and share joint first authorship.
